# TELD with limited foraminoplasty has potential biomechanical advantages over TELD with large annuloplasty: an in-silico study

**DOI:** 10.1186/s12891-021-04504-1

**Published:** 2021-07-10

**Authors:** Jingchi Li, Chen Xu, Xiaoyu Zhang, Zhipeng Xi, Mengnan Liu, Zhongxin Fang, Nan Wang, Lin Xie, Yueming Song

**Affiliations:** 1 Department of Orthopedic Surgery and Orthopedic Research Institute, West China Hospital/West China School of Medicine for Sichuan University, 37# Wuhou Guoxue road, Chengdu, Sichuan Province 610041 P.R. China; 2 Department of Spine Surgery, Changzheng Hospital Affiliated to the Naval Medical University, Shanghai, 200041 China; 3 Department of Spine Surgery, Affiliated Hospital of Integrated Traditional Chinese and Western Medicine for Nanjing University of Chinese Medicine, Nanjing, Jiangsu Province 210028 P.R. China; 4 Macau University of Science and Technology, Macau, 999078 China; 5 Fluid and Power Machinery Key Laboratory of Ministry of Education, Xihua University, Chengdu, 610039 China

**Keywords:** Biomechanical deterioration, Transforaminal endoscopic lumbar discectomy, Endoscopic dynamic drill, Facetectomy, Iatrogenic annulus injury

## Abstract

**Background:**

Facetectomy, an important procedure in the in–out and out–in techniques of transforaminal endoscopic lumbar discectomy (TELD), is related to the deterioration of the postoperative biomechanical environment and poor prognosis. Facetectomy may be avoided in TELD with large annuloplasty, but iatrogenic injury of the annulus and a high grade of nucleotomy have been reported as risk factors influencing poor prognosis. These risk factors may be alleviated in TELD with limited foraminoplasty, and the grade of facetectomy in this surgery can be reduced by using an endoscopic dynamic drill.

**Methods:**

An intact lumbo-sacral finite element (FE) model and the corresponding model with adjacent segment degeneration were constructed and validated to evaluate the risk of biomechanical deterioration and related postoperative complications of TELD with large annuloplasty and TELD with limited foraminoplasty. Changes in various biomechanical indicators were then computed to evaluate the risk of postoperative complications in the surgical segment.

**Results:**

Compared with the intact FE models, the model of TELD with limited foraminoplasty demonstrated slight biomechanical deterioration, whereas the model of TELD with large annuloplasty revealed obvious biomechanical deterioration. Degenerative changes in adjacent segments magnified, rather than altered, the overall trends of biomechanical change.

**Conclusions:**

TELD with limited foraminoplasty presents potential biomechanical advantages over TELD with large annuloplasty. Iatrogenic injury of the annulus and a high grade of nucleotomy are risk factors for postoperative biomechanical deterioration and complications of the surgical segment.

## Background

The discovery of the Kambin triangle [1] was rapidly followed by the enhanced use of transforaminal endoscopic lumbar discectomy (TELD) for the treatment of lumbar disc herniation (LDH) [2]. Facetectomy is an important procedure in the in–out and out–in techniques of TELD [3–5]. The procedure involves foramen enlargement via the removal of part of the superior articular process (SAP) and ligamentum structures [6–8]. Facetectomy is useful for expanding the surgical field and decompressing the exiting nerve root, especially in patients with foramen stenosis [4, 9–12].

The zygapophyseal joint (ZJ) guides spinal motion, transfers a substantial amount of the compressive load and bending and shearing moments (i.e. limits excessive motion) and protects structures in the functional spinal unit (FSU) [13–20]. Pathological changes in spinal load distribution may occur after high-grade facetectomy, resulting in injury to the surgical FSU, which is a risk factor for symptom recurrence and disc degeneration [16, 21–23]. The ZJ is an important structure for maintaining spinal stability [13, 14, 24]. Instability in the surgical segment caused by facetectomy is a risk factor for biomechanical deterioration, which results in degeneration of the surgical FSU and poor long-term prognosis [19–22, 25]. These deductions are consistent with the findings of our published finite element (FE) numerical studies [26–28], which demonstrated that a higher grade facetectomy might be associated with biomechanical deterioration and lumbar instability; these changes may be related to further degeneration and symptom recurrence [27–29]. Because axial rotation could enhance the vulnerability of the posterior annulus and the ZJ could restrict lumbar spinal motion under axial rotation, iatrogenic injury of the SAP in TELD may also increase the risk of annulus tear, recurrence of related symptoms and acceleration of disc degeneration [17, 18, 24, 30].

**Fig. 1:**
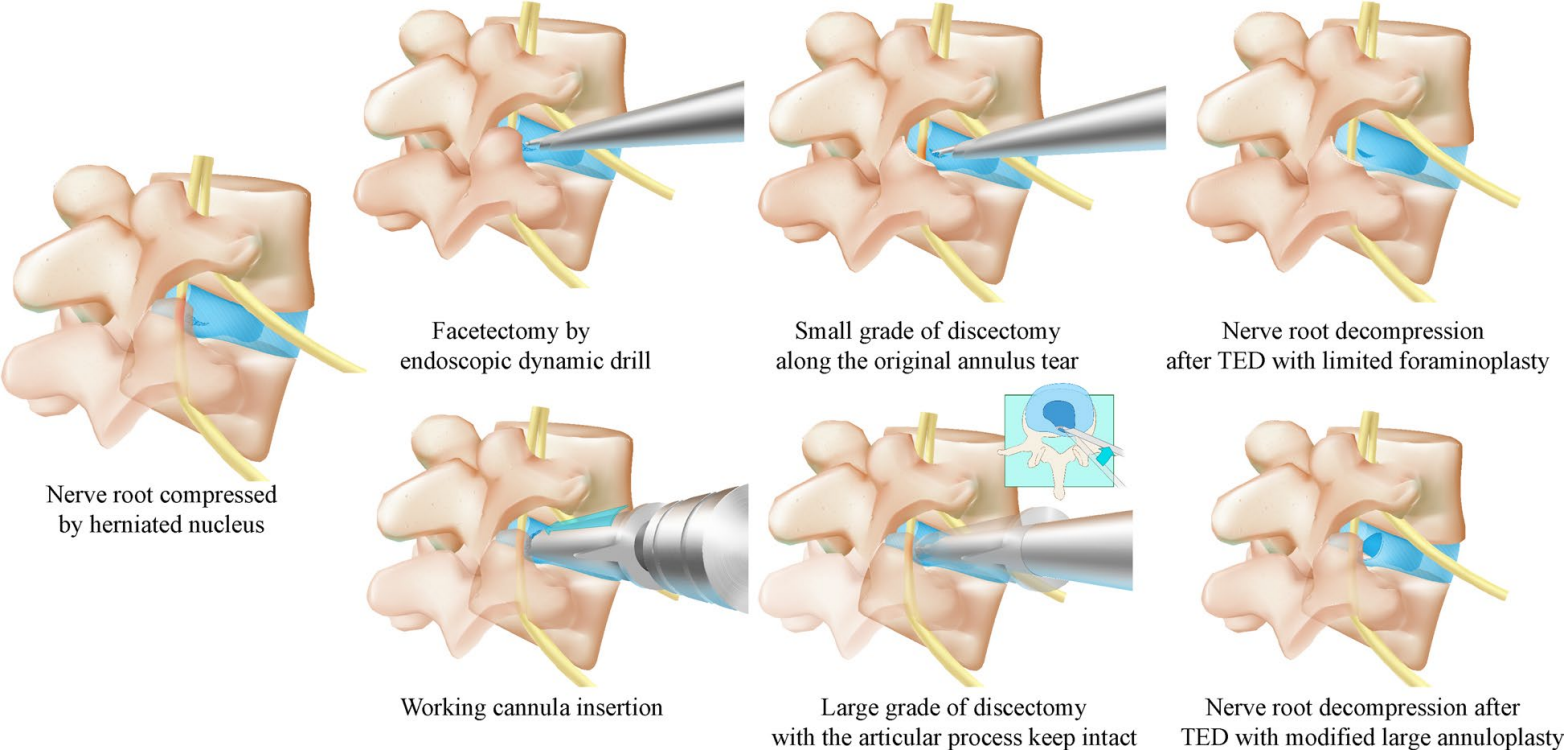
Schematic diagram of the optimisation of TELD via two strategies (ligamentum structures are not indicated for brevity)

The standard in–out technique could be modified to avoid facetectomy in TELD for patients without foramen stenosis. In this modification, hereafter referred to as large annuloplasty, the cannula is inserted into the disc space via the Kambin triangle. The herniated disc is then removed without damage to the SAP (i.e. without facetectomy) by pressing down on the cannula and using different sizes of bending forceps. This technique avoids the related risks of biomechanical deterioration and postoperative complications (Fig. 1).

Despite the benefits of modification, however, the optimised in–out surgical strategy retains its original defects, which may also lead to poor clinical outcomes. Specifically, the risk of recurrent lumbar disc herniation (RLDH) has been reported to increase dramatically with the expansion of the annulus tear (i.e. by over 25% when the annulus tear is larger than 6 mm) [31–35]. The strength of the scar tissue in the outer lamellae of the annulus is insufficient to prevent RLDH [36, 37]. Considering that the diameter of our working cannula is 7.5 mm (Type WTS127502, Joimax International, Irvine, CA, USA) and its insertion would inevitably lead to iatrogenic injury of the annulus, a higher grade of nucleotomy in the modified in–out TELD technique appears to be necessary to prevent RLDH (Fig. 1) [3]; unfortunately, this surgical strategy also presents limitations.

Preservation of the residual nucleus postoperatively is important to maintain spinal biomechanical function [19, 38, 39]. A high grade of nucleotomy may lead to the pathological distribution of stress in the annulus and render the latter vulnerable to fatigue damage under cyclic loading [38–41]. Such damage may result in annulus tears, which could accelerate disc degeneration. Disc collapse could also be accelerated by this pathological process [16, 39, 40], and the risk of lumbar instability may increase on account of the laxity of soft tissues. The incidence of foramen stenosis could increase as a result of the decrease in foramen cross-sectional area (CSA) following the collapse of the surgical FSU without facetectomy [24, 25, 42]. Hence, a higher incidence of symptom recurrence may be observed in patients with higher grades of nucleotomy [16, 23]. The collapse of the surgical segment and disc degeneration may also lead to irregular secondary spinal load transmission, which has been proven to increase the load of the ZJ cartilage and the risk of ZJ osteoarthritis (ZJOA), hypertrophy of the articular process and spinal stenosis [43–45]. Thus, a higher grade of nucleotomy, the remedial action prescribed to reduce the risk of RLDH caused by iatrogenic annulus tears in the in–out technique, may lead to greater risks of poor clinical outcomes and low satisfaction in patients after TELD [32].

**Fig. 2:**
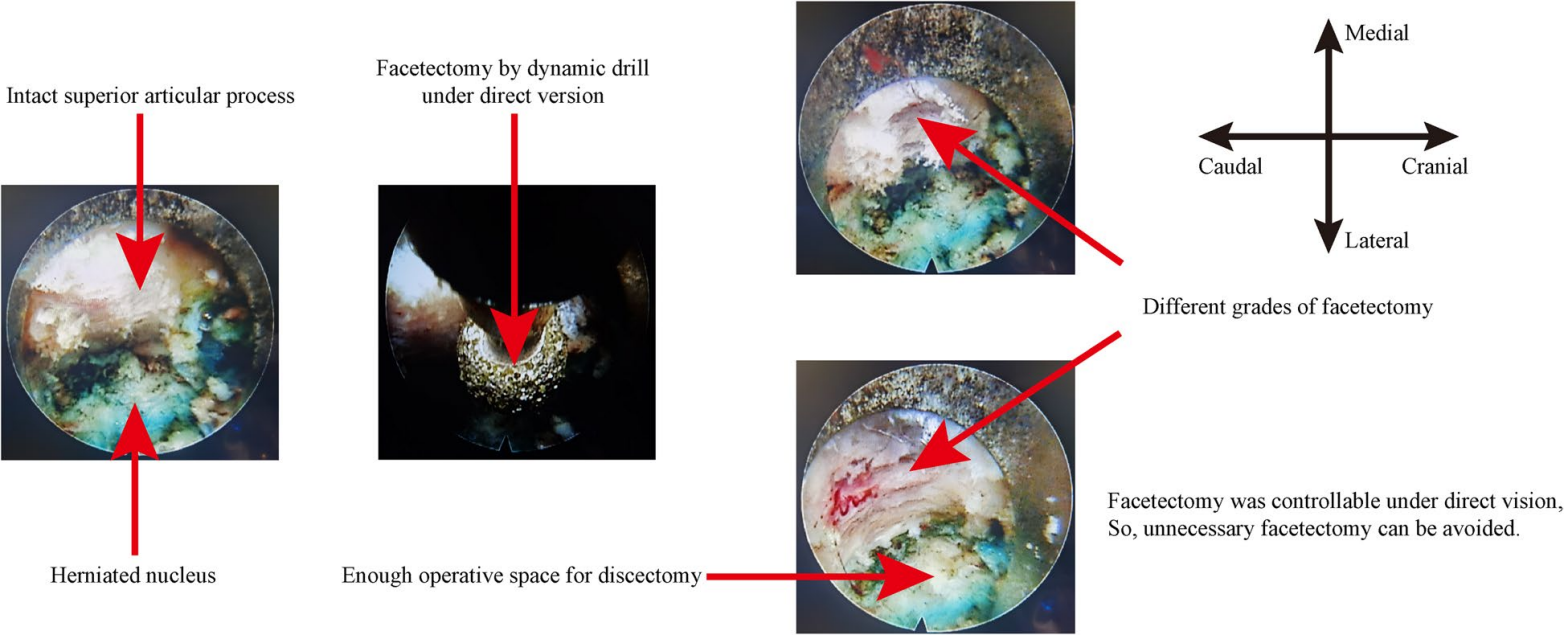
Endoscopic view of precise facetectomy by a dynamic drill

A high grade of nucleotomy as a remedial procedure for iatrogenic annulus injury is not usually necessary in the out–in technique of TELD. If the size of the original annulus tear is less than 6 mm, the residual annulus tissue is not generally expected to lead to serious clinical symptoms [46]. Nucleotomy could be accomplished along the original annulus tear without any iatrogenic annulus injury. Discectomy can be accomplished in patients with the contained type of LDH (i.e. LDH with an intact annulus) by using bipolar radiofrequency to produce a small slit (i.e. less than 6 mm) in the annulus; in this case, higher grades of nucleotomy are unnecessary. Facetectomy may be limited by endoscopic dynamic drill for which could be accomplished precisely under direct version with its assistance. In our clinical practice, we successfully restricted the grade of facetectomy to less than one-third for patients without foramen stenosis and protected the ZJ cartilage and capsule (Fig. 1 and Fig. 2). More importantly, the controllable risk of postoperative spinal instability and biomechanical deterioration after endoscopic nucleotomy with a low grade of facetectomy was proven in our published studies [26–28].

Having established the above theoretical and practical foundation, we hypothesise that, even if an iatrogenic injury of the SAP could be avoided, compared with TELD with large annuloplasty, the modified out–in technique of TELD, hereafter referred to as limited foraminoplasty, presents potential biomechanical advantages. This hypothesis was verified by examining the biomechanical effects of TELD with large annuloplasty and TELD with limited foraminoplasty via validated three-dimensional lumbo-sacral models. LDH patients are often middle-aged or elderly, and degenerative changes in their original discs may have potential impacts on the postoperative biomechanical environment [23, 47, 48]. Herein, surgical simulations and FE analysis were accomplished using models with and without degeneration. To the best of our knowledge, this research is the first to provide real evidence clarifying these issues.

## Methods

### Model construction

**Fig. 3:**
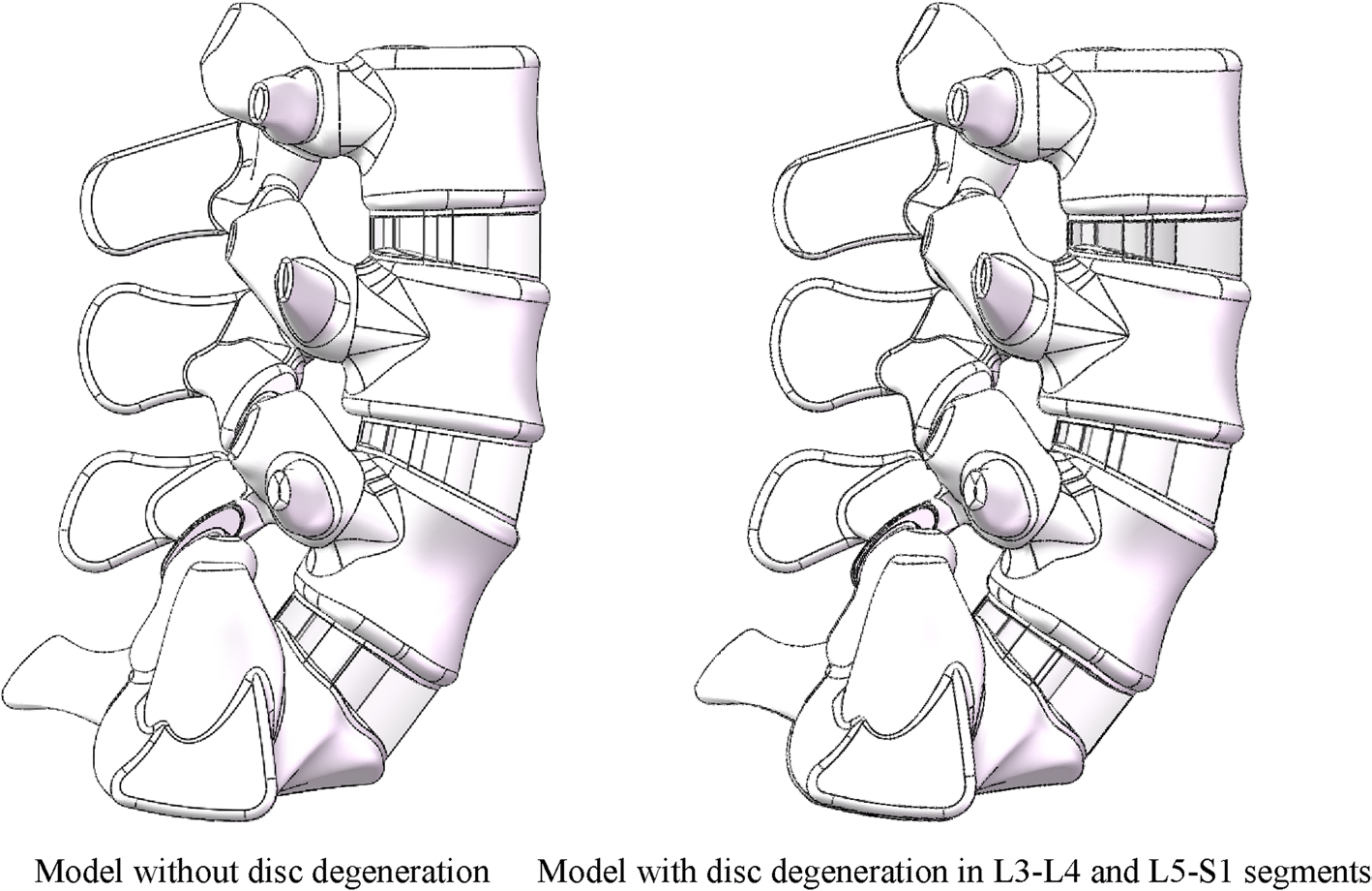
Intact 3D models constructed in the current study

An intact FE model of L3–S1 was constructed in our published studies [26, 28, 49]. The bone structures in this model included cortical, cancellous and posterior structures and the nonbony components included intervertebral discs and ZJ cartilages. The IVD consisted of the nucleus core, the surrounding annulus and cartilage endplates [50, 51], and the thickness of the cortical structures and endplates was set to 0.8 mm [47, 48, 52]. Ligaments and ZJ capsules were constructed by cable elements [49, 53]. Facet cartilages were defined by surface–surface contact elements, and the gap between cartilages was set to 0.5 mm [47, 54]. In the model of disc degeneration in segments adjacent to the surgical segment, the disc height was reduced to 67%, the CSA of the annulus was increased by 40% and the material properties of the annulus and nucleus were modified according to previously published studies (Fig. 3) [23, 47, 48].

### Boundary and loading conditions

The intact models were set to be symmetric in the sagittal plane to increase their computational efficiency by allowing the unilateral calculation of the bending and axial rotation loading conditions [27]. Different sizes of hybrid elements were established in the FE models, and the mesh was refined as thin structures and structures with large deformation [27, 49, 55]. A mesh convergency test was performed on the intact models by evaluating the change in maximum annulus shear stress to ensure the computational credibility of a model; the model was considered to be converged if the change in computational value was less than 3%. All degrees of freedom were fixed under the S1 inferior, stress and moments were applied to the L3 superior [49, 56] and the contact between facet cartilages was considered frictionless [53, 56].

### Model calibration and validation

During model calibration, the stiffness of the ligamentum structures was slightly modified within the physiological range to reduce differences between the computed biomechanical indicators and those described in widely cited in-vitro studies [50, 56–61]. The reliability of the calibrated model was then ensured by conducting multi-indicator model validation, which was accomplished by comparing the computed range of motion (ROM), intradiscal pressure (IDP) and disc compression (DC) with the results of previous in-vitro studies under different loading conditions [62–65].

### Simulation of TELD with large annuloplasty and which with limited foraminoplasty

**Fig. 4:**
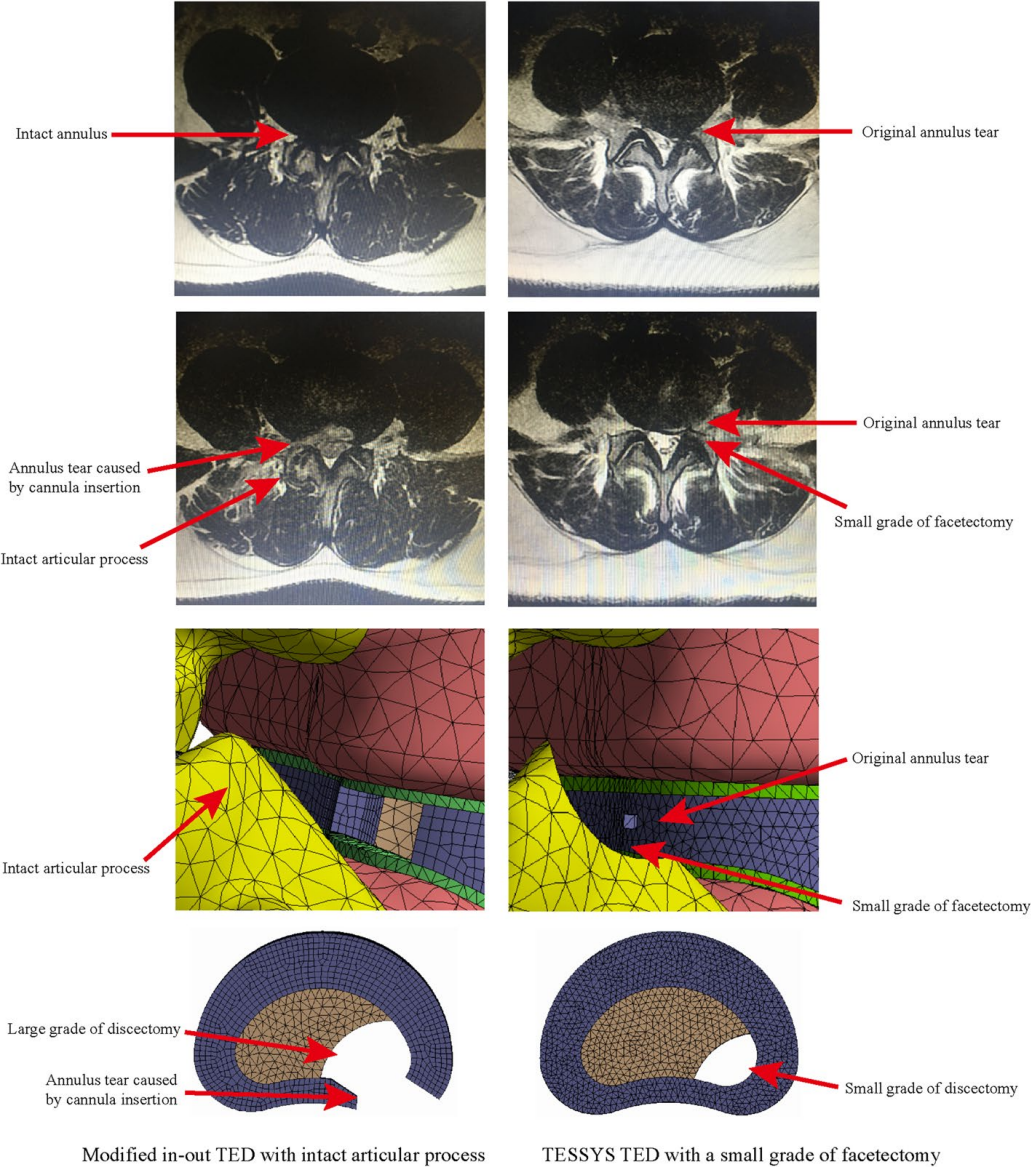
Surgical model construction and the corresponding imaging data

The right side of segment L4–L5 was selected to simulate TELD. TELD with limited foraminoplasty was simulated according to a reported surgical technique and our clinical experience [4, 49]. In brief, a 3 mm long and 1 mm wide incision was made on the annulus to simulate the annulus tear. One-sixth of the nucleus around the incision was removed to simulate a low-grade nucleotomy. The vertex of the facetectomy was located on the cranial tip of the SAP, and one-third of the SAP and ligamentum flavum (LF) was excised (Fig. 4) [6, 7, 26, 49]. The simulation of TELD with large annuloplasty and intact SAP was conducted by setting the original annulus tear as the centre of working cannula insertion. The surrounding 7.5 mm area of the annulus was completely deleted to simulate iatrogenic injury. One-third (i.e. twice the range of nucleotomy in TELD with limited foraminoplasty) of the nucleus around the annulus tear was removed to simulate a high-grade nucleotomy (Fig. 4). Pathological changes caused by DD were simulated in segments L3–L4 and L5–S1, and the surgical simulation was kept consistent between models with and without DD (Fig. 4). The FE models constructed in this study were named Models 1–6 to simplify the discussion (Table 1).

**Table 1:** Summary table of named FE models

	Models without disc degeneration	Models with adjacent segments disc degeneration
Intact model	1	4
**TELD models with large annuloplasty**	2	5
**TELD models with limited foraminoplasty**	3	6

## Results

### Model validation

**Fig. 5:**
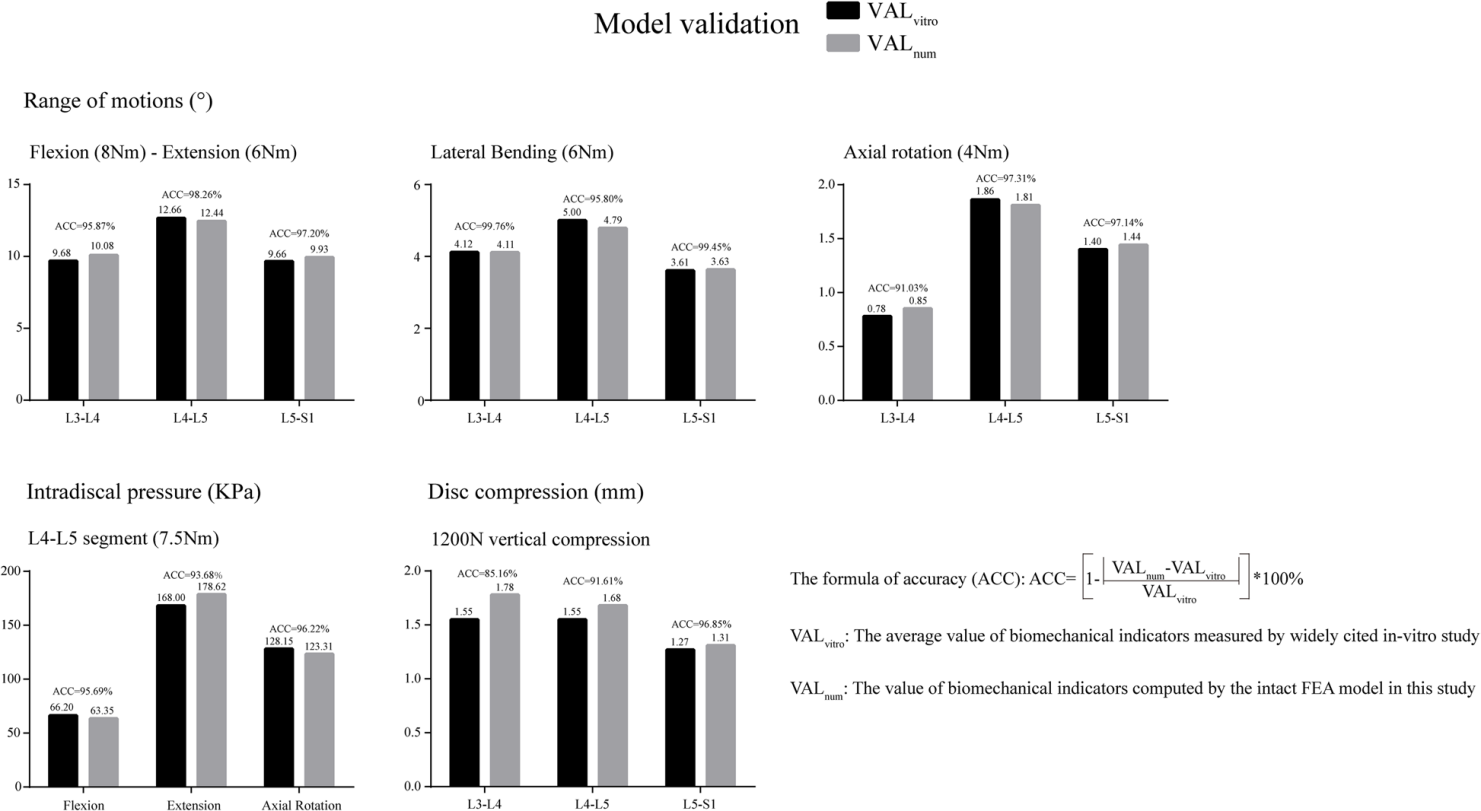
Model validation

The computational accuracy (ACC) of the models is presented in Fig. 5. In this study, the ACC for all parameters determined, except for DC in segment L3–L4, exceeded 90%. The ACC of DC in segment L3–L4 was 85.2%, and the difference between our computational result and the average value determined from an in-vitro study was clearly less than one standard deviation [58]. Moreover, DD in segments adjacent to the surgical segment led to slight increases in IDP and decreases in facet contact force (FCF) in the surgical segment, which is consistent with published studies [23, 48]. Thus, we believe that our models represent the actual biomechanical environment well.

### Biomechanical change in different models

The concept of FCF is emphasised here. FCF was not recorded during flexion because cartilages under this loading condition were not in contact. Besides, cartilages in the bending side were in contact, and the opposite side of cartilages were in contact in the axial rotation condition. In other words, FCF under left lateral bending is observed on left-side cartilages, whilst FCF under left axial rotation is observed on right-side cartilages and vice versa.

**Fig. 6:**
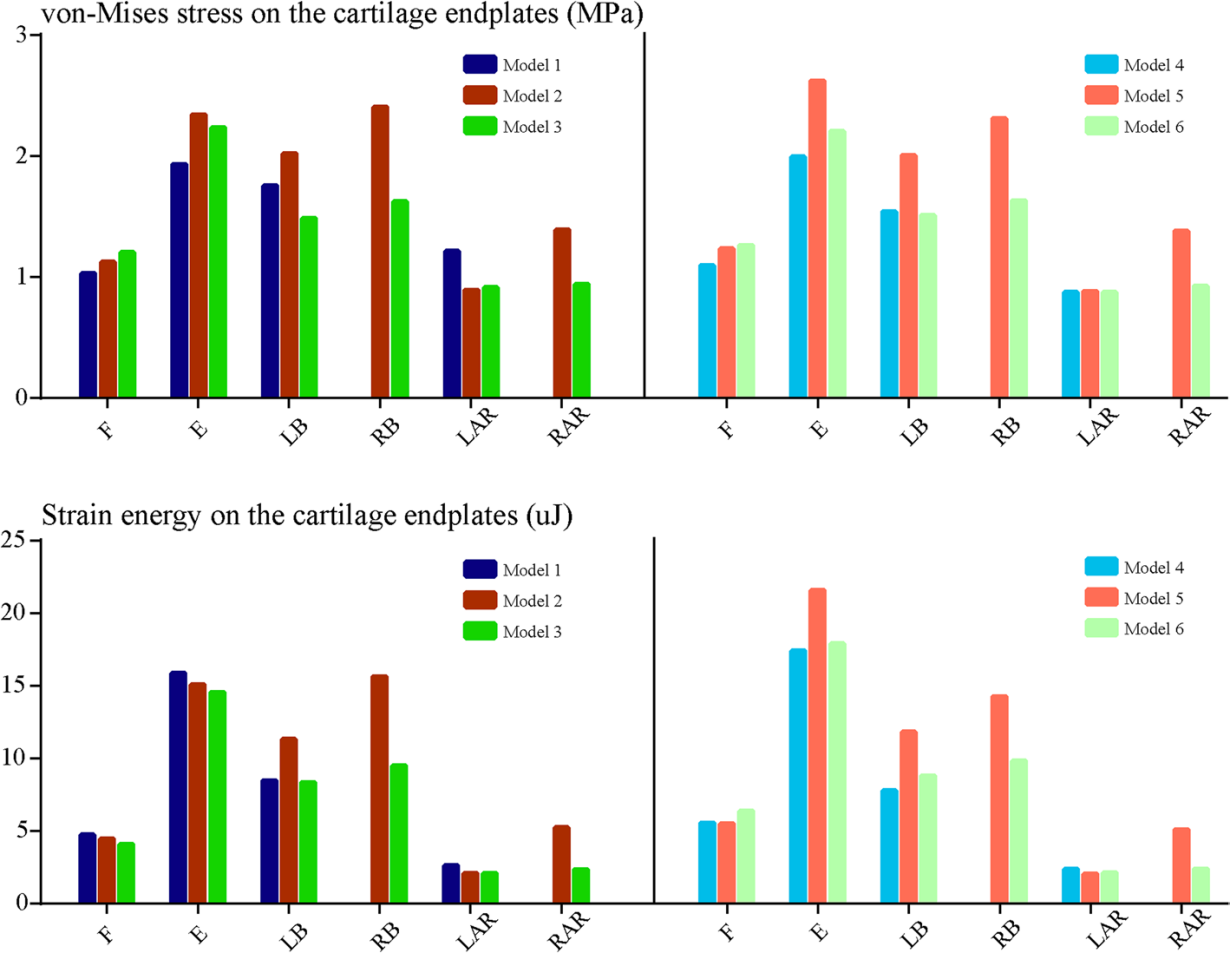
Variations in biomechanical indicators related to ‘endplate-type’ disc degeneration. F: flexion, E: extension, LB: left bending, RB: right bending, LAR: left axial rotation, RAR: right axial rotation. The descriptions of Models 1–6 are provided in Table 1

**Fig. 7:**
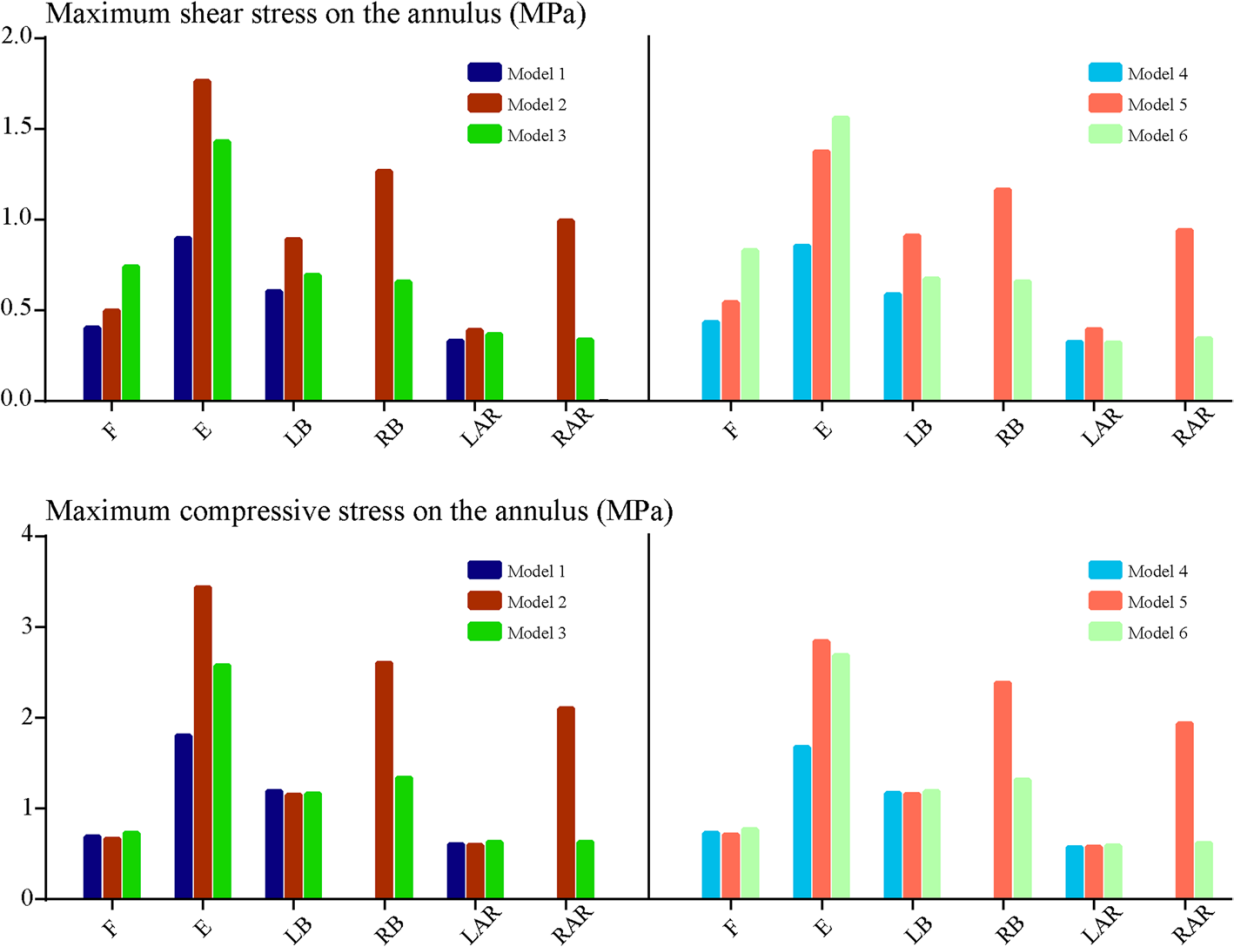
Variations in biomechanical indicators related to ‘annulus-type’ disc degeneration

**Fig. 8:**
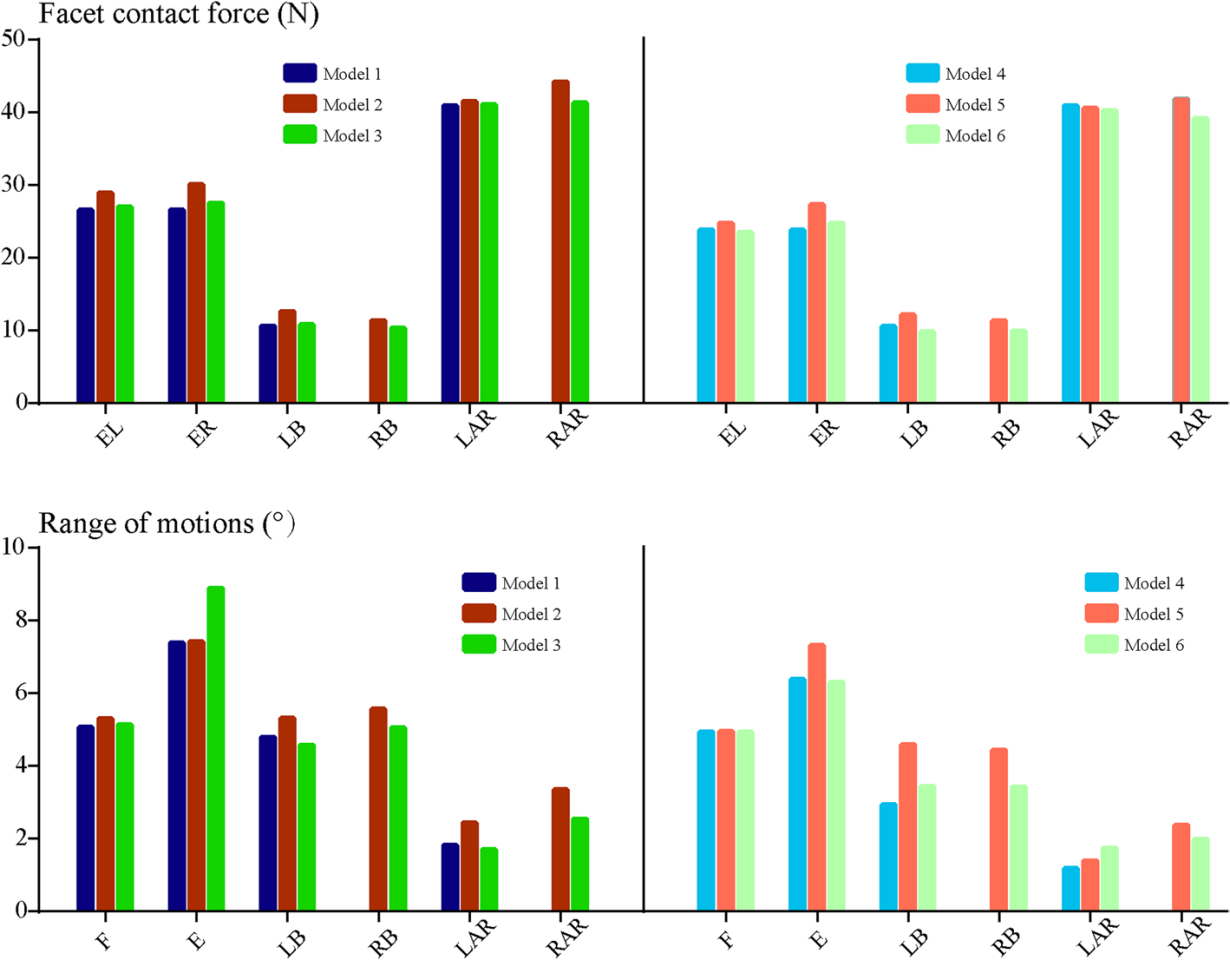
Variations in biomechanical indicators related to ZJ degeneration and lumbar instability

**Fig. 9:**
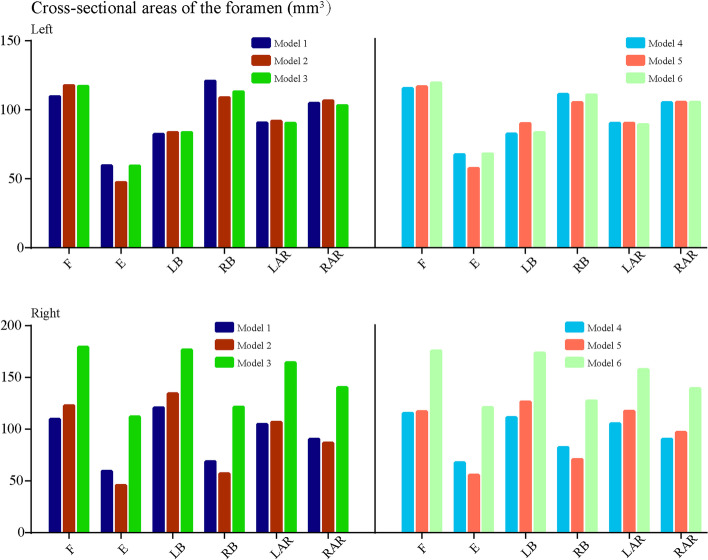
Variations in CSA

Biomechanical indicators related to the acceleration of DD, ZJOA, segmental instability and spinal stenosis in the surgical segment were computed and recorded (Figs. 6, 7, 8 and 9). Compared with the intact models, the models simulating TELD with limited foraminoplasty showed slight degenerative changes under most loading conditions, whilst the models simulating TELD with large annuloplasty revealed obvious biomechanical deterioration. Most of the biomechanical indicators in the surgical segment, except for FCF and ROM, deteriorated with DD in adjacent segments, and the change trends of the original biomechanical parameters in postoperative models did not vary or clearly increase in the degenerated models.

Obvious biomechanical changes were observed during bending and rotation to the surgical side (i.e. right lateral bending and axial rotation). Specifically, a difference of over 40% in maximum von Mises stress and strain energy was observed on the cartilage endplates during right bending and rotation, and the difference in cartilage strain energy under right rotation exceeded 100% in different postoperative models (Fig. 6). A difference in maximum shear and compressive stress of over 100% was noted under the condition of right rotation (Fig. 7). Whilst the overall trend was identical to those of the above indicators, changes in FCF and ROM in the different models were relatively minor (Fig. 8). Foraminoplasty could obviously enlarge the area of the foramen in the surgical site, and the area in TELD models with large annuloplasty was even smaller than preoperative models under extension and right bending conditions (Fig. 9).

Obvious biomechanical changes can be observed in bending and rotation conditions to the surgical side (i.e. right lateral bending and axial rotation). Specifically, greater than 40% difference in maximum von-Mises stress and strain energy on the cartilage endplates can be observed in right bending and rotation conditions, and the difference in the cartilage strain energy under right rotation was even more than 100% in different postoperative models (Fig. 6). Besides, more than 100% difference in the maximum shear and compressive stress was evaluated under right rotation condition (Fig. 7). In contrast, while the overall trend was the same as the above indicators, the changes of FCF and ROM in different models were relatively minor (Fig. 8). Additionally, foraminoplasty could obviously enlarge the area of foremen in the surgical side, and which in TELD models with large annuloplasty was even smaller than preoperative models under extension and right bending conditions (Fig. 9).

## Discussion

### Objective of this study

This work evaluated the risks of biomechanical deterioration and postoperative complications in the surgical segment caused by TELD with large annuloplasty and intact SAP and TELD with limited foraminoplasty and without annuloplasty. Intact lumbo-sacral models with and without disc degeneration and the corresponding models obtained after the investigated operations were performed were constructed, and biomechanical indicators closely related to lumbar degenerative diseases were computed and evaluated. The importance of the biomechanical environment for achieving positive postoperative clinical outcomes has been repeatedly demonstrated [23, 49, 51]. Thus, investigations on the biomechanical changes caused by two different surgical techniques of TELD are of great significance for optimal surgical strategy selection.

### Notable points in the model construction process

Adjacent segments, rather than the surgical segment itself, were selected to construct the DD models. This model construction strategy is based on our clinical experience. As mentioned earlier, DD is very common in TELD patients. This nature of degenerative change may not always lead to serious clinical symptoms but could adversely affect the biomechanical environment in adjacent segments [23, 47, 48]. Hence, simulations of disc degeneration are meaningful for the evaluation of real postoperative biomechanical environments. Disc collapse during DD could lead to reductions in the CSA of the Kambin triangle, and the risk of exiting nerve root injury in a degenerated disc increases during the insertion of the working cannula without facetectomy [66, 67]. As such, LDH with a narrow disc space may be considered a contraindication for the application TELD with large annuloplasty, and the surgical segment was excluded during the construction of degenerative change models.

Although ZJ degeneration has been closely related to DD [43, 45, 57, 68] and some FE studies have constructed ZJ degenerative models by reducing the facet gap [47, 56], in this work, we abandoned the construction of ZJ degeneration. The gap thickness of the ZJ should reflect the cartilage wear, sclerosis and hyperplasia of subchondral bone [59, 69, 70], but these pathological processes are difficult to simulate during model construction. Specifically, decreasing the facet gap by increasing the thickness of the facet cartilage is completely contrary to the pathological changes accompanying ZJ degeneration. Besides, if the gap is reduced by increasing the thickness of the bone tissue of the articular process, the definitions of material properties for sclerotic subchondral bone structures, which obviously differ from those of normal bone tissues, become inaccurate [15, 71, 72], and the casual definition of material properties without reliable data will reduce the credibility of this study. Hence, we chose to construct DD models without a change in facet gap [48, 56].

The grades of facetectomy in the out–in TELD models and discectomy in the in–out TELD models were set as one-third, consistent with the maximum value we have observed in our clinical practice. This modelling strategy was selected because facetectomy and nucleotomy were previously assumed to be the main reasons behind the poor clinical outcomes obtained after these operations. Therefore, higher grades of these two procedures could lead to more pronounced biomechanical deterioration and provide a clearer reference for evaluating these techniques.

### Clinical significance of biomechanical indicators

Disc collapse and DD acceleration in the surgical segment and the resulting secondary pathological changes are the most significant causes of poor clinical outcomes in patients following non-fusion lumbar surgery [33, 73, 74]. As reported by Adam et al., the injury of the endplates and annulus may be considered two different pathways in the DD process [21]. The maximum von Mises stress and strain energy of the endplates were recorded to evaluate the risk of DD caused by endplate lesions and ossification. Endplates play a key role in pressure distribution. Postoperative abnormal stress concentration on the endplates increases the risk of lesions in these structures [21, 75, 76] and may result in inflammatory responses, autoimmune reactions and disc innervation, all of which are considered significant triggers for DD acceleration and increased risk of lower back pain (LBP) [50, 77–79].

IVD is an avascular structure, and the most important pathway for its metabolism is trans-endplate diffusion [80, 81]. According to Wolff’s law, the concentration of strain energy, a type of compensatory reaction to endplate stress concentration, may be considered a predictive factor for IVD ossification [82, 83]. Occlusion of the trans-endplate diffusion pathway could lead to DD acceleration [43, 79, 84, 85]. Endplate injury caused by abnormal stress concentration is closely associated with the disruption of the annulus and may be reflected by the deterioration of the biomechanical indicators of this structure, especially in its post and post-lateral parts [21, 29, 81]. The concentration of shear and compressive stresses has been proven to be related to different types of annulus tears and the resulting DD, thereby resulting in discogenic LBP and RLDH [22, 23, 84]. Hence, we can speculate that the above biomechanical indicators are credible predictors for the assessment of postoperative prognosis.

Foramen stenosis is another vital reason behind the deterioration of clinical outcomes. Special attention should be paid to models after in–out TELD with intact SAP because the risk of foramen stenosis increases with disc collapse caused by a higher grade of discectomy in the surgical segment without foraminoplasty [73, 74, 86]. An increase in FCF is a risk factor for not only cartilage wear and the resulting degenerative osteoarthritis of the ZJ [15, 44, 72], a trigger of LBP [22, 25, 45], but also foramen stenosis because larger loads could promote osteogenic activity [43, 82, 87]. More importantly, disc collapse and degeneration of the surgical segment lead to pathological stress concentration on the ZJ cartilages, resulting in degenerative osteoarthritis and osteophyte formation [15, 45]. Lumbar instability, which has been proven to be related to LBP and further DD, is an important cause of deterioration after non-fusion surgery [85, 88, 89]. Therefore, ROM can be used as an indicator not only for model validation but also for the assessment of postoperative complications, and close interactions were observed amongst different biomechanical indicators.

Biomechanical deterioration can generally be observed in models with DD. Although DD in adjacent segments did not obviously exacerbate biomechanical deterioration in the surgical segment, the vicious cycle of DD could be observed; this finding highlights the significance of this FE study from a novel perspective [21, 90]. Specifically, in DD, the deterioration of the biomechanical environment caused by inappropriate surgery may be continuously amplified and lead to a devastating prognosis. Therefore, the selection and optimisation of a surgical technique based on a biomechanical FE study are of great significance.

The risk of endplate calcification, annulus tears and the resulting DD in the surgical segment may be remarkably accelerated in patients after TELD with large annuloplasty, and the potential risk they present to surgical segment instability, ZJOA and the compression of existing nerve roots should be taken into consideration. The biomechanical advantages of TELD with limited foraminoplasty indicate that the risk of these complications is generally lower in patients treated with this surgical method.

These speculations are consistent with previously published biomechanical reports. Specifically, nucleus removal could lead to the concentration of von Mises and compressive stresses on the annulus, particularly on the posterior and post-lateral rings of the structure, resulting in an increased risk of developing annulus microfractures and disc collapse [38, 91, 92]. In-vitro studies have noted an increase in FCF at denucleated segments [93]. These biomechanical and morphological changes have been reported to be initial triggers for symptom recurrence and poor prognosis in patients [32, 94].

### Limitations

Firstly, ligaments were constructed by cable elements, and simulation of LF excision was accomplished by reducing the foramen CSA. Cable elements cannot stimulate the folding, hypertrophy and calcification of ligaments, and these pathological changes have been reported to be vital risk factors for spinal stenosis and nerve compression.

The proliferation of scar tissue on the annulus and its biomechanical effects cannot be evaluated; this issue is fairly common in FE studies. Considering that the size of annulus breakage is an important variable in this study, biomechanical changes brought about by the formation of annulus scar tissue, the strength of which cannot stop RLDH, may also exert potential biomechanical effects on patient prognosis. Hence, the current computational results should be interpreted with an awareness of this defect. Follow-up clinical studies are recommended to obtain more definitive conclusions.

## Conclusions

Biomechanical deterioration can be observed in in–out TELD models with large annuloplasty and without facetectomy. Annuloplasty caused by the insertion of the working cannula and a high grade of nucleotomy to prevent RLDH may result in poor clinical outcomes for this surgical method. Modified out–in TELD with limited foraminoplasty appears to provide considerable biomechanical advantages.

## Data Availability

All the data of the manuscript are presented in the paper.

## Abbreviations

ACC: Accuracy
CSA: Cross-sectional areas
DC: Disc compression
FCF: Facet contact force
FE: Finite element
IVD: Intervertebral discs
IDP: Intradiscal pressures
LDD: Lumbar degenerative diseases
LDH: Lumbar disc herniation
LBP: Low back pain
LF: Ligamentum flavum
RLDH: Recurrent lumbar disc herniation
ROM: Range of motions
TELD: Transforaminal endoscopic lumbar discectomy
SAP: Superior articular process
YESS: Yeung endoscopic spine system
ZJ: Zygapophyseal joint
